# Disability Inclusive Youth (DIY) Research: an innovative and co-creative study to improve inclusion of children and youth with disabilities in health research in East Africa: a mixed-method study protocol

**DOI:** 10.12688/wellcomeopenres.24916.2

**Published:** 2026-02-14

**Authors:** Femke Bannink Mbazzi, David John Musendo, Gatera Fiston Kitema, Joyce Muhenge Olenja, Andrew Sentoogo Ssemata

**Affiliations:** 1 Disability Research Group, MRC/UVRI and LSHTM Uganda Research Unit, Entebbe, POBOX 49, Uganda; 2 International Centre for Evidence in Disability, London School of Hygiene and Tropical Medicine Faculty of Epidemiology and Population Health, London, England, Keppel Street, WC1E 7HT, UK; 3 Lifetime Research Group, Muhazi, Rwamagana, Eastern Province, PO Box 3029, Rwanda; 4 University of Rwanda School of Medicine and Pharmacy, Gikondo, Kigali City, P.O. Box 4285, Rwanda; 5 Department of Public Health, University of Nairobi Faculty of Health Sciences, Nairobi, Nairobi County, PO BOX 30197 - 00100, Kenya

**Keywords:** Youth, disability, health research, co-creation, co-design, capacity building, inclusion, Africa

## Abstract

Young people with disabilities are rarely included in research, yet 75% of the East African population is under 30 years old and an estimated 15% have a disability. Without including young persons with disabilities in designing and implementing health research, we will not achieve global development goals. The aim of Disability Inclusive Youth (DIY) study is to explore barriers and facilitators to inclusion of children and youth with disabilities in health research, co-create solutions to make health research in East Africa disability inclusive, and create a disability knowledge and research centre to inform and support inclusive health research in the region. The DIY study will develop a novel participatory approach to enhance the inclusion of children and youth with disabilities in health research in East Africa. We will build capacity of 12 youth with disabilities from Uganda, Kenya, and Rwanda through a research training programme. The youth researchers will conduct interviews with 75 leading health researchers, 30 key health stakeholders, and 60 children and youth with disabilities and their caregivers. Together with the youth we will design recommendations to make health research more inclusive of children and youth with disabilities through participatory workshops. At the end of the study we will have established a regional knowledge and research centre where children and youth with disabilities contribute to and health researchers consult about disability inclusive health research. Data collection methods include survey questionnaires, in-depth interviews, case studies, video and photovoice. Data will be analysed qualitatively using a thematic approach in NVIVO and quantitatively using STATA. This is a 5 year study funded by Wellcome, through the London School of Hygiene and Tropical Medicine, in partnership with the MRC/UVRI & LSHTM Uganda Research Unit, the University of Nairobi and the University of Rwanda / Lifetime Research Group.

## Plain language summary

Young people with disabilities are rarely included in research, yet 75% of the East African population is under 30 years old and an estimated 15% have a disability. Without including young persons with disabilities in designing and implementing health research, we will not achieve global development goals. This study will forge a novel participatory approach to include children and youth with disabilities in health research in East Africa. We will build the research capacity of 12 youth with disabilities in Uganda, Kenya and Rwanda to explore the barriers and facilitators to disability inclusive health research. The youth researchers will interview leading health researchers, key health stakeholders, government and research organisations, health service providers and children and youth with disabilities. Together with the youth we will establish recommendations to make health research more inclusive of children and youth with disabilities.

## Introduction

Disability is a contested, complex, dynamic, and multidimensional concept that has not yet been explored sufficiently in the African context. ^1^ There is a need to value diverse understandings and discourses to avoid generalized and simplified descriptions of disability experiences. ^2– 9^ Globally, young persons with disabilities are left behind in access to healthcare, education, social participation, and employment. ^10– 14^ Youth with disabilities are less likely to access health services compared to their non-disabled peers. ^15, 16^ The COVID-19 pandemic has further exacerbated the exclusion and poor health outcomes of youth with disabilities. ^17– 19^

Much of the East African population is under 30 years of age: 78% in Rwanda and Uganda and 75% in Kenya. Disability statistics are lacking and underestimate the worldwide prevalence of disability of 15%, ^20^ with 12.4% in Uganda, ^21^ 2.2% in Kenya, ^22^ and 5.9% in Rwanda. ^23^ Children and youth rarely participate in the design and implementation of health research and argue for further efforts ‘to incorporate children with a broad range of impairments, drawing on theory and methodology from disability and childhood studies’. ^24, 25^ Understanding the experiences of young people with disabilities and their engagement in leading and shaping research is key to developing and ensuring contextually appropriate and effective strategies to promote participation and inclusion. ^26, 27^

In addition, considering intersectionality, ^28– 30^ and Afrocentrism, ^30^ in disability and health research is key. Important intersections with disability and health are the notion of *Ubuntu* as a key element in African disability discourse, ^1, 30^ poverty, which is closely related to disability in a complex, non-linear, and non-circular way, ^31^ as well as stigma. ^32^ and the rural-urban divide. ^33^

### Rationale

In the Disability Inclusive Youth (DIY) study, we plan to develop research capabilities and leadership skills, as well as support and train East African researchers with disabilities to undertake high-quality research and promote a positive and inclusive culture from an African disability studies discourse. Social and peer support have been highlighted as a key aspect in enhancing social inclusion and health related quality of life for youth with disabilities in Africa. ^16, 34, 35^ *Ubuntu* (‘I am because we are’) informed the *Obuntu bulamu* intervention designed with children with disabilities to improve participation and inclusion in communities. ^34^ In this intervention, children were paired with their peers to become agents of change. In Africa, peer-to-peer support has been successfully utilized in parental support programs for children with disabilities, ^36^ as well as HIV prevention and treatment programs for youth. ^37– 39^ In addition, this study will utilize the lessons learned from the *Disabled Youth Investigates research collaboration* in which youth with disabilities were offered research training and internships in pairs in Uganda, ^40^ based on the good training practices of the Zimbabwean Youth Research Academy. ^41^

In the DIY study, we will scale up the youth researchers’ training. ^40^ by involving youth as co-researchers in conducting research for a period of three years in the three countries. We will build on lessons learned from participatory research, including photo and video voice and action workshops with children and their peers. ^42– 44^ For dissemination, we will utilize our experience of participatory film making from the *Obuntu bulamu* and *Young Africa Works* studies, in which youth with disabilities co-created short films about their participation in education and employment in Uganda, Kenya, and Rwanda.

Through a peer to peer support approach based on *Ubuntu* (‘I am because we are’) values we will train and mentor African youth with disabilities in health research, conceptualize and measure inclusion of children and youth with disabilities in health research in Kenya, Rwanda and Uganda, and develop guidelines for best practice on including children and youth with disabilities in health research in the region. Framed through the concept of *Ubuntu* persons with disabilities are seen as part of humanity and interrelated to others with mutual responsibilities. This approach entrenches people with disabilities within communities, reduces isolation, and improves participation and inclusion.

### Aims and research questions

This study aims to explore barriers and facilitators to the inclusion of children and youth with disabilities in health research, co-create solutions to make health research in East Africa disability inclusive, and create a disability knowledge and research centre to inform and support inclusive health research in the region.

This study has three main components: 1) co-creative innovative participatory disability health research with youth with disabilities to understand the barriers and facilitators of inclusion of children and youth with disabilities in health research, 2) co-design solutions to make health research inclusive of children and youth with disabilities, and 3) capacity building of research teams and co-production and sharing of knowledge with youth with disabilities.

The DIY research questions are:

1. What are the barriers and facilitators for health researchers to include children and youth with disabilities in health research?
2. What are the experiences of children and youth with disabilities and their caregivers in participating in health research?
3. How can equitable and inclusive health research be ensured for children and youth with disabilities?
4. What are the experiences of persons with disabilities in conducting health research in East Africa?
5. How can African disability knowledge be made available to advance health research in the region?

### Study approach

**Figure 1.**
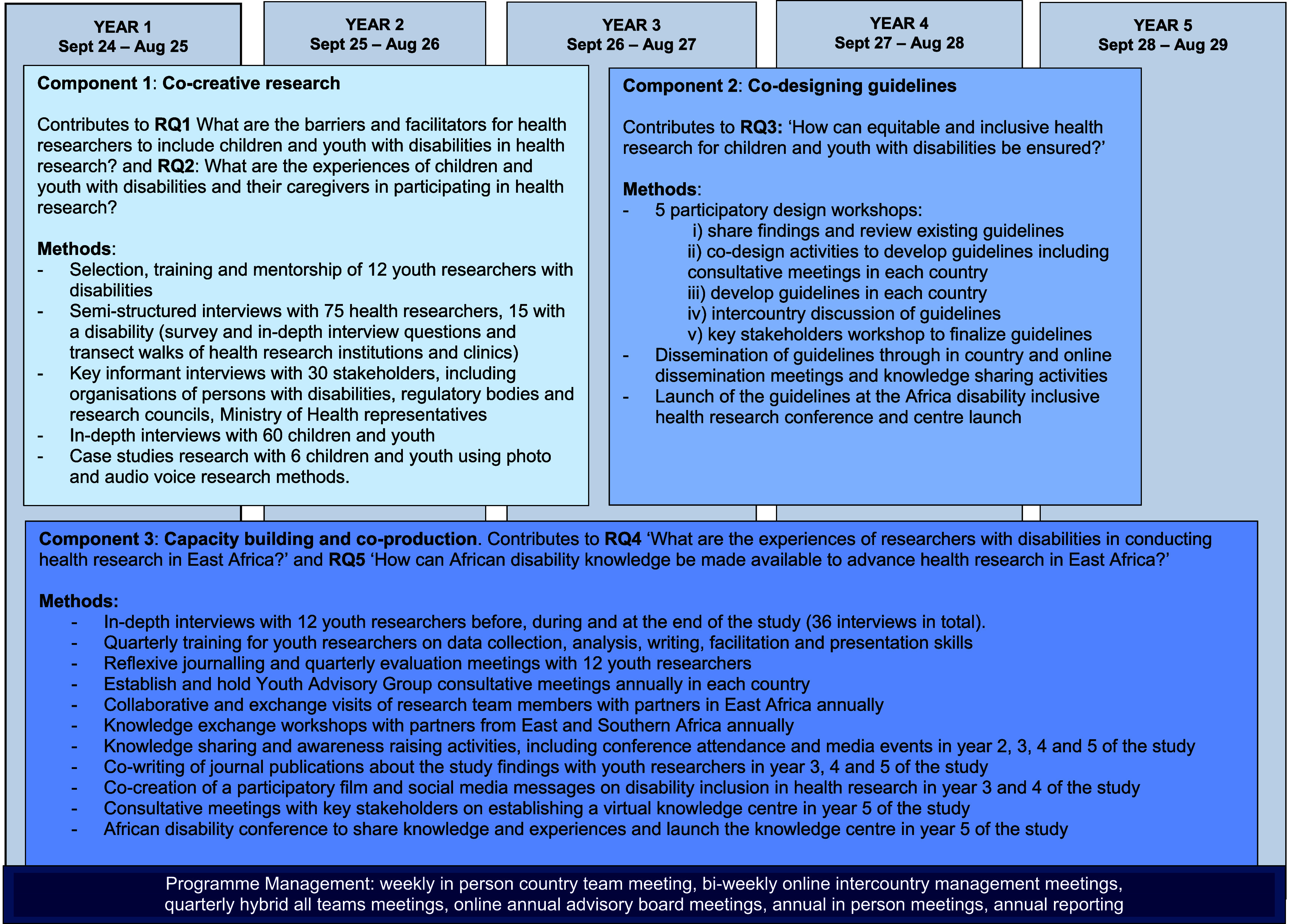
Study component, research questions and methods overview.

This study aims to generate shifts in understanding how health research in low- and middle-income countries can become more inclusive of children and young people with disabilities. It will develop methodologies and tools that promote disability inclusion and benefit health research by developing research capabilities and leadership skills among East African researchers with disabilities. Figure 1 shows the study components, research questions and data collection methods over the 5 years of the study.

***Component 1: Co-creative innovative participatory disability health research with youth with disabilities***

We will select 12 young people with disabilities in Kenya (4), Rwanda (4), and Uganda (4) through an open and competitive selection process. Job adverts will be shared through the organizations websites and social media platforms, universities, organizations of persons with disabilities and youth with disabilities within the research organisations’ existing networks.

All 12 youth researchers will receive a 5-day in person training in Uganda, followed by a one week training in their respective countries. Co-investigators from the three countries will co-facilitate the training with members of the existing youth advisory group and graduates from the *Disabled Youth Investigates research* project. ^40^ The training will cover principles of collaboration, ethics, safeguarding, informed consent, quantitative and qualitative research methods, data analysis, and community engagement. Teaching methods will include lectures, group work, reflective exercises, role plays and evaluation activities. After the 5 days, the youth researchers will have an orientation week at their respective research institutes and will complete Good Clinical Practice and General Data Protection in Research trainings and prepare for their internships.

Youth will undergo practical research training within ongoing health research projects at in-country research institutes under the mentorship of a researcher for three months. After completing their training and internship period, the 12 youth researchers will be trained on the study protocol, interview guide design, identification, screening, consenting of study participants, and will be mentored in conducting qualitative interviews by observing senior researchers and practicing under supervision. They will receive training on taking field notes, storing and transcribing data. Youth researchers will participate in monthly mentoring and feedback meetings with their respective mentors to reflect on learning and address challenges faced including possible power imbalances. They will keep reflexive journals and will receive further training over the course of the study, e.g. in coding in NVIVO, thematic analysis, writing reports, validating findings, facilitation and presentation skills. The youth will receive specific training on interviews techniques and ethics and safeguarding in data collection with children and youth.

To answer Research Question 1, ‘What are the barriers and facilitators for health researchers to include children and youth with disabilities in health research? *’* semi-structured interviews will be conducted with 75 health researchers and 30 key stakeholders in Kenya, Rwanda, and Uganda.

To answer Research Question 2, ‘What are the experiences of children and youth with disabilities and their caregivers in participating in health research?’ in-depth interviews with 60 children and youth with disabilities (and were appropriate their caregivers) will be conducted in Kenya, Rwanda, and Uganda. Six case studies will be conducted.

Details on the selection of study participants and data collection methods are outlined below.

***Component 2: Developing guidelines and best practices to improve inclusion of children and youth with disabilities in health research***

To answer Research Question 3, ‘How can equitable and inclusive health research for children and youth with disabilities be ensured?’ we use an innovative and participatory approach to formulate guidelines and best practices for health researchers in East Africa and beyond.

The development process will include five days of participatory workshops co-led by youth researchers, three in each country and two multi-country workshops. Workshop participants (approximately 20 per country) will include youth researchers, research team members, selected youth advisory members and study participants. In the first workshop, the youth will discuss the findings of the interview data collection in relation to barriers and facilitators that health researchers face and experience children, and youth with disabilities have had in participating in health research in East Africa. The research team will support the youth researchers to review and describe existing guidelines and best practices in disability inclusion in sub-Saharan Africa and ask them to brainstorm on activities that could be illustrative in the guidelines to improve the inclusion of children and youth in health research in the future. Through a participatory action design on the 2 ^nd^ and 3 ^rd^ workshop days, the youth will co-create guidelines and best practices that may consist of consultation with youth with disabilities, community discussions, awareness-raising activities on participation in health research, and other activities designed by the youth. Youths will document the process through photos and video voice. In a 4 ^th^ workshop, 12 youth researchers from the three countries will come together to design a multi-country guideline and best-practice document. This document will be shared with key stakeholders for input and discussion in 5 ^th^ workshop and consultative meeting, which will be a hybrid meeting to allow inputs from stakeholders from all countries. Key stakeholders will include leading disability health researchers, national research councils, national organizations of persons with disabilities, and research and ethics committees. Adaptations will be made based on feedback from the participants and youth researchers. The final guidelines will be shared with health researchers, the Ministry of Health, Research Council, and other key stakeholders in the three countries through dissemination meetings and will be made available online through partner websites.

***Component 3: Capacity building and co-production and sharing of knowledge***

To answer research question 4, ‘What are the experiences of researchers with disabilities in conducting health research in East Africa?’, in-depth interviews with 12 youth health researchers with disabilities in Kenya, Rwanda, and Uganda will be conducted before, during, and at the end of the study. These interviews will be conducted by a doctoral student who will evaluate the youth researchers experiences over the course of the study. Attention will be paid to power imbalances. In addition, the Youth Advisory Groups and the 12 youth researchers will be asked to answer research question 5: ‘How can African disability knowledge be made available to advance health research in East Africa?’ through reflexive journaling, consultative meetings and participatory workshops. Furthermore, consultative meetings will be held with disability researchers from the region to inform them how best to create a virtual knowledge centre for health researchers to co-design research with youth with disabilities. This may include the establishment of a consultative group or a pool of youth with disabilities who researchers can work with in co-designing grant applications and find potential advisory members and researchers with disabilities for health research programs.

Throughout the study, we will carry out activities to build the capacity of the next generation of researchers and facilitate the co-production of knowledge and shared learning with youth with disabilities in East Africa. Activities will include a) collaborative and exchange visits of team members with regional partners in East Africa; b) knowledge exchange workshops with partners from East and Southern Africa, including leading disability researchers from collaborating and other universities and research institutes in the region; c) involvement of youth in knowledge sharing and awareness raising activities on disability inclusion in research, such as national and international conferences and webinars, community awareness-raising events, social media messaging, participation in local and national radio or tv-programs, meetings with organizations of persons with disabilities, stakeholders, and policy makers among others; d) co-writing of journal publications about the study findings; and e) co-creation of a participatory film and social media messages with youth with disabilities about the study, which will target persons with disabilities and key stakeholders in the three countries. This will be disseminated through partner websites and social media channels, as well as disabled persons’ organizations in the three countries; f ) organization of an African disability conference in which we will invite disability scholars, organizations of persons with disabilities, NGO, and government representatives from East and Southern Africa to share knowledge and experiences in an in-person conference in Uganda.

### Study design

This longitudinal cohort study will utilize a novel co-creative participatory mixed-method implementation research design, which collects in-depth information on barriers and facilitators to the inclusion of children and youth with disabilities in health research in Kenya, Rwanda, and Uganda.

75 health researchers, 15 with disabilities, will be interviewed at the start of the study and answer a survey questionnaire. They will be selected from academic and research institutions in Kenya, Rwanda, and Uganda. A purposive sampling method will be used, enrolling 75 health researchers consecutively until the sample size is reached. Fifteen health researchers with disabilities will be purposively selected using snowball sampling. The representation of gender and impairment categories (physical, visual, hearing, cognition, communication, and multiple) will be ensured as much as possible. The interview guide and survey questionnaire will be developed with the youth researchers. Key areas of focus in the interviews will be the researchers’ perceptions of and experience with disability inclusive research, including barriers and facilitators in study design and implementation in the research organizations they work in. The survey questionnaire will include questions on number of persons with disabilities recruited in ongoing health research, the type of research tools used, and measure accessibility of study sites and research organisations’ office space utilizing existing accessibility audit observation tools and transect walks.

Sixty youth with disabilities (approximately 20 per country) who participated in past and ongoing health research will be recruited through health research, academic institutions, and key stakeholders in the three countries mentioned above. The representation of age (approximately 20 per age category: 0–8 years, 8–17 years, 18–30 years), gender and impairment categories (physical, visual, hearing, cognition, communication, and multiple) will be ensured. The sample size is based on recommendations for qualitative studies and the expected data saturation of groups of youth with different types of impairments. Interview guides will be developed with the youth researchers and will be informed by the findings from the health researchers and key stakeholder interviews. Youth researchers will conduct the interviews with children and youth with guidance from research team members. Key areas of focus will include the experiences children and youth (and their caregivers) had in health research, including recruitment processes, participation in study activities, accessibility of research facilities and reasonable accommodations. Interviews will be audio-recorded and where involving people who are deaf, video recorded if conducted in sign language. Depending on the age and impairment of the child or youth we will draw on different participatory methods, including the use of drawings. A caregiver may be involved as a proxy or assistant in interviews, in instances where an individual age or impairment severely affects their ability to understand or communicate their experiences. Caregiver voices will be reported separately from children and youth voices.

From the in-depth interviews, six youths, currently participating in a health research study, will be purposefully selected and invited to participate in photo and video voice data collection to narrate health research experiences, using cameras provided by the program. They will be paired with two youth researchers and trained on photo and video voices and asked to document their experiences in health research, such as clinic visits and intervention activities they participate in at home, at the health facility, or in the community over a period of three months. The training will address privacy-sensitive image, video, and audio capture, including guidance on avoiding identifiable third parties and minimising disclosure of disability status and research participation. Participants will be instructed not to record identifiable individuals without consent and to reframe, anonymise, or exclude content where consent cannot be obtained. Throughout the three-month documentation period, weekly follow-ups, conducted by the youth researchers, will be used to review materials, reinforce ethical practices, and address potential privacy or disclosure risks. Participants will retain full control over their materials and will approve any images, videos, or narratives before they are shared beyond the research team. The youth researchers, with the support of the research teams, will follow up with the youth involved on a weekly basis to back up the visual data collected and document the youths’ narratives around the photo and video data collection.

To develop guidelines and share best practices based on the study findings, five participatory workshops will be held: three to design the guidelines in every country and create social media messages and story boards for the film, and two multi-country meetings to finalize the document and related messages. The youth will be introduced to a Kenyan Ugandan filmmaker who created participatory films with research participants in earlier studies. Together with the filmmaker, youth researchers will develop storyboards based on the study findings and document the best practices. The photo and video case study participants will be invited to participate in the co-creation of the film. The design process of the guidelines too will be documented by the filmmaker. In addition, a consultative meeting will be held with the youth and stakeholders to discuss how best to share the guidelines and to set up a knowledge centre where youth with disabilities offer African disability knowledge and experiences to researchers and co-design future health research.

In addition, the youth recruited as youth researchers and their mentors will be interviewed to evaluate the youth co-research approach before, during and at the end of the program. Furthermore, they will keep reflexive journals throughout the project to document their experiences.

### Study population and participant recruitment

Youths with disabilities (n=12) will apply for the training and mentorship program through a competitive process and will be selected through an interview and group task process in which reasonable accommodations will be provided. The criteria for application will include: a) aged 18 – 30 years; b) self-identify as having a disability and have a moderate to severe impairment or disability (the Washington Group Short Set of questions will be used as a screening tool) or have a disability as defined by the country’s national disability council; c) youth should be a university graduate or at least have a diploma post-secondary A-level; and d) be interested and available to participate in the training and co-research with remuneration. Equal numbers of male and female youth will be selected. The study follows the SAGER guidelines on sex and gender in research studies. ^43^

The 75 health researchers, of whom 15 with a disability, will be sampled from various research organizations and entities in the three countries. Research leaders, managers and implementers engaged in health research in academic and other research organisations will be interviewed. Key stakeholder interviews (30) will be held with representatives of regulatory bodies, national research councils, national councils for persons with disabilities, organizations of persons with disabilities, relevant government departments and civil society partners working in disability and health.

Youth study participants (n=60) will be identified in collaboration with the research teams in the field locations, health research institutes, organizations of persons with disabilities (e.g., National Councils for Persons with Disabilities), civil society, local leaders and community partner organizations, with snowballing applied as needed. Particular attention will be paid to underrepresented and less networked youth. Participants will be purposively sampled to ensure representation from all genders, different ages, socioeconomic class, urban/semi-urban/rural, and impairment types (physical, hearing, vision, intellectual, mental health, and multiple). Inclusion criteria are: a) aged 0–17 for children and 18 – 30 years for youth; b) self-identify as having a disability and have a moderate to severe impairment or disability (the Washington Group Short Set of questions will be used as a screening tool) or have a disability as defined by the country’s national disability council; c) is a study participant in an ongoing health research study or has participated in a health research study in Kenya, Rwanda or Uganda in the past 5 years; d) able to verbally or non-verbally express themselves in an interview, or has a caregiver who can provide by proxy information for children under 8 and children and youth aged 8 – 30 with severe communication difficulties; e) interested in participating in co-designing and participation in research; and f ) resident in Kenya, Rwanda, or Uganda.

### Data collection

The following data collection is planned within this study.

- Seventy five semi-structured interviews with health researchers at research institutes, academic institutions, and national research councils in Kenya, Rwanda, and Uganda (approximately 25 in each country), of which fifteen semi-structured interviews with health researchers with a disability (approximately five in each country)
- Sixty in-depth interviews with children and youth with disabilities (and where appropriate) their caregivers who participated in health research studies in Kenya, Rwanda, and Uganda (approximately 20 in each country).
- Thirty key stakeholder interviews with organizations of persons with disabilities, administrators of health research organizations and universities, ethical review boards, and national research councils (10 per country).
- 6 case studies of children and youth with disabilities using photo and video voice to narrate experiences of participating in health research studies (2 per country).
- 36 in-depth interviews with youth researchers with disabilities in Kenya, Rwanda, and Uganda will be conducted before (12), during (12), and at the end (12) of the programme.
- 54 participatory workshop design workshops to develop disability inclusive research guidelines.
- 1 consultative meeting to inform the development of the centre of excellence and conference.

Data collection and analysis followed the COREQ and SRQR criteria for qualitative research. ^45, 46^

### Data outputs

This project will produce the following outputs: 1) 201 transcripts from recorded interviews with research participants; 2) field notes and reflexive journals of the youth researchers; 3) 100 photos and short video clips of six participants and a 10 minutes participatory film; 4) participatory workshop data from five meetings; and 5) evaluation data from 60 questionnaires and interviews. The transcripts, field notes, and workshop notes will be produced in Microsoft Word. Photos and video files will be produced in. jpg and .mp4 formats.

Data documentation will include a data dictionary on questionnaire responses, transcript annotations that indicate processing performed (e.g., to anonymize), and other information necessary to understand the context.

### Data management and storage

A dedicated data and compliance manager for the program will ensure data management and storage for all sites. A study database will be designed and managed according to the guidelines of the Uganda National Council for Science and Technology, the Kenyan National Council for Science and Technology, the Rwanda Data Protection Law (2021), international GDPR guidelines, and the principles of ICH-Good Clinical Practice guidelines compliant with SOPs to ensure data security. The databases will be designed to quality control the data at entry and have logic checks, and data will be backed up twice a day by the programmers and once a week by the data manager. Each site/country will have separate access to the database, and personal identifiers will not be shared across sites.

The original consent and data forms will be stored in locked files at each partner institution. Handheld data collection devices (tablets or phones) will be used to collect and enter quantitative data, which will be connected to the program’s REDCAP database. Qualitative data will be recorded, transcribed, and stored on a computer that is password protected. Where sign language is used, video recordings will be made instead of audio recordings with the consent of the participants. Specific attention will be paid to quality assurance and continuous job mentoring for newly trained youth coresearchers.

Data will be stored, preserved, and remain accessible for a minimum of 10 years after the research is concluded.

### Data sharing

Data sharing will follow international GDPR guidelines and country-specific protocols and guidelines, including Wellcome’s Open Data and Accessible Source Materials Guidelines for Humanities and Social Sciences.

All program staff will undergo training on data privacy and security protocols to reinforce compliance and safeguard participant information throughout the study. Personal identifying data will not be shared across sites, and the data will be managed according to the data protection laws for each country.

Data will be made available through the LSHTM Data Compass ( https://datacompass.lshtm.ac.uk/) and assigned a Digital Object Identifier (DOI) within 6 months of study completion or prior to relevant publications (whichever is sooner). Description metadata describe the information content, collection context, and information on how it may be accessed and reused. Data that cannot be fully anonymized, such as transcripts that contain indirect identifiers, are shared through an application process (controlled access).

### Data analysis

The data analysis will include triangulation of interviews, case studies, and visual data. Qualitative data collected through interviews, focus group discussions, and participatory methods will be transcribed verbatim and, where necessary, translated into English to ensure consistency in analysis across study sites. Transcripts will then be uploaded into NVivo 14, a qualitative data management and analysis software, to support the systematic coding and organization of the data. A thematic analysis approach was used to identify, analyze, and report patterns (themes) within the data, following Braun and Clarke’s six-phase framework: (1) familiarization with the data through repeated reading of transcripts; (2) generation of initial codes to capture meaningful features of the data; (3) searching for themes by clustering related codes; (4) reviewing and refining themes for coherence and distinctiveness; (5) defining and naming themes to capture their essence; and (6) producing the final report, linking thematic findings to the study objectives. ^42^ Inductive and deductive coding strategies will be used to ensure that the analysis captures both emergent insights and issues related to the study’s conceptual focus on disability inclusion. Youth researchers will actively participate in the transcription and coding of qualitative data, review, and discuss emerging themes to enhance reflexivity, deepen interpretation, and ensure that the analysis reflects the lived experiences and perspectives of children and youth with disabilities.

Quantitative data analysis will begin with basic descriptive statistics to summarize the demographic characteristics of the participants, including age, sex, disability type, education level, and geographic location. These analyses will provide an overview of the composition of the study population across the three countries Uganda, Kenya, and Rwanda. We will also generate frequency tables and cross-tabulations to explore patterns in the inclusion of persons with disabilities in health research programs, including whether and how disability was considered in the study design, participant recruitment, data collection tools, and dissemination practices. Where appropriate, comparisons will be made across countries, institutions, and researcher characteristics to identify trends, gaps, and good practices for disability inclusion. The analysis also assesses the extent to which existing health research programs have adopted inclusive methods, such as accessible communication materials, reasonable accommodations, and involvement of children and youth with disabilities in research planning and implementation. All analyses will be conducted using Stata statistical software, and the findings will inform the development of practical guidelines and policy recommendations for disability-inclusive health research in East Africa.

### Dissemination

Dissemination activities will involve publications in peer-reviewed journals, presentations at conferences, and stakeholder meetings in communities.

It is not anticipated that this research will generate data that require intellectual property protection. Any training materials and film that the youth will co-produce may be published on the LSHTM website with explicit participant consent. Participant contributions will be recognized where identification is allowed and agreed with the participants.

### Community engagement and involvement

Community engagement and involvement (CEI) is a key component of this study, in line with the World Health Organization’s core principle of Health 2020: reducing health inequities across the population as well as the importance of participation and responsiveness with the full engagement of people.

Youth with disabilities from an existing Youth Advisory Group in Uganda were included in the design of this study. Youth advisory members are representatives of youth organizations for persons with disabilities, nominated by the National Unions of Disabled Persons in Uganda and the National Council for Persons with Disabilities. The advisory group consists of 12 youth with different impairments. We asked the youth advisory members to identify the need for further research in a group meeting and shared a draft outline of the study protocol for inputs. In addition, we asked young interns, staff, and students with disabilities in Kenya, Rwanda, and Uganda, as well as youth with disabilities who participated in the Young Africa Works study to provide insights into the areas of focus and how they might would like to be involved in this study.

Youth advisory and existing youth with disabilities working at partner institutes will be involved in this study through regular advisory and staff meetings. In Uganda, the existing Youth Advisory Group will be asked to volunteer participants for the advisory meetings specific to the DIY study. In addition, youth representatives who participated in the *Disabled Youth Investigates* project that informed the design of the DIY study, ^40^ will be invited. In Kenya and Rwanda, Youth Advisory Group members will recruited in consultation with the National Council for Persons with Disabilities and organizations of persons with disabilities. The organizations will be asked to nominate members who represent a diverse range of impairments, age groups, and genders.

Annual advisory meetings and dissemination meetings will be led by youth with disabilities.

### Ethical considerations

This study has been approved by the ethical review committee of the London School of Hygiene and Tropical Medicine (Ref: 32274/RR/37804), as well as the respective ethics committees in the three countries: Uganda Virus Research Institute (Ref: GC/127/1054) and the Uganda National Council for Science and Technology (SS3665ES), University of Nairobi/Kenyatta National Hospital Ethics and Scientific Review Committee (Ref: KNH-ERC/A/244), Kenyan National Council for Science, Technology and Innovation, and the Rwanda National Ethics Committee (No 342/CMHS IRB/2025).

An information sheet will be provided to the participants during recruitment and reiterated verbally as part of the informed consent process. As international and national disability practice forms will be made accessible to persons with disabilities, we will read out information sheets and consent forms for persons with visual impairments and/or difficulties in reading. All information sheets and consent forms are available in English, Luganda, Kiswahili, Kinyarwanda, and Braille. A sign language interpreter will be employed to provide consent to participants with hearing impairments. Simplified information sheets will be available to participants with cognitive or communication difficulties. Written consent will be obtained from all participants by signing or thumbprinting in the presence of an impartial witness. For participants with difficulties in communication or understanding, assent will be sought together with written consent from their caregivers. Reasonable accommodations will be offered during each phase of the study to both the staff and participants with disabilities. This may include hiring personal assistants or sign language interpreters, providing a quiet space, as well as captioning and screen reading software and specialized computer equipment to ensure youth researchers can do their work effectively.

All staff and youth researchers will be trained in research ethics and safeguarding. The project has a safeguarding policy with a triage system and country specific referral list. Participants who disclose their experience of violence, are at risk of violence, and/or present with other acute health difficulties will immediately be referred to specialized health care providers and protection services. The study team members, including youth researchers will report safeguarding concerns to the country’s study coordinator, who will report and refer as necessary. The study coordinator will work closely with partner organizations to ensure that referrals of participants are responded to in an appropriate and timely manner and are flexible and sensitive to a range of disabilities. The study coordinators will inform the country team leads who will inform the principal investigator and file adverse event reports with the relevant regulatory authority.

The research teams will hold debriefing meetings with the youth researchers after conducting interviews and support the youth in case they encounter distressing narratives. Where needed, youth researchers can be referred for external counselling support. The youth researchers will be employed as staff members of the respective research organizations and will benefit from health insurance and other services provided for research staff in each country.

## Discussion

This study responds to the growing global and regional imperative to make health research more inclusive, particularly for youth with disabilities. Despite increased attention to disability rights and inclusion in development and health policies, ^44^ young people with disabilities remain underrepresented in both the design and participation of health research, especially in low- and middle-income countries. ^24, 25^ This lack of inclusion not only perpetuates health inequities but also limits the relevance, responsiveness, and effectiveness of research intended to improve health outcomes. ^26, 27^

The study has the following expected outcomes:

1. In-depth knowledge of the barriers and facilitators health researchers experience in including children and youth with disabilities in Uganda, Kenya, and Rwanda.
2. In-depth knowledge of the experiences of children and youth with disabilities and their caregivers in health research in Uganda, Kenya, and Rwanda.
3. Generated and shared guidelines for the inclusion of children and youth with disabilities in health research in East Africa.
4. 12 youth with disabilities trained in health research, contribute to making health research disability inclusive in Uganda, Kenya and Rwanda
5. Established a regional knowledge and research center where children and youth with disabilities contribute to, and health researchers can consult about disability inclusive health research.

The study has the following strengths and limitations:

- The study employs a co-creative participatory design involving youth with disabilities as co-researchers, enhancing contextual relevance and empowerment.
- The inclusion of diverse settings (Kenya, Rwanda, and Uganda) and a range of stakeholders (researchers with and without disabilities, youth with disabilities, caregivers, and policy actors) allows for rich comparative insights and broader applicability of findings.
- The integration of interviews, survey questions, observations, case studies, participatory workshops, photos, and video voices allows for triangulation and a deeper understanding of lived experiences and systemic barriers.
- Newly trained youth co-researchers may have varied research experience, which could affect consistency and depth in data collection and analysis across sites. To mitigate this, we planned to enhance supervision and mentorship.
- A longitudinal and multi-component design, involving diverse data types (video, interviews, workshops) and countries, may pose challenges in maintaining methodological consistency and timelines. Inbuilt regular team meetings and mentoring will attempt to reduce this risk.

## Data Availability

No data are associated with this article.
