# Supplementary material for: Disability Inclusive Youth (DIY) Research: an innovative and co-creative study to improve inclusion of children and youth with disabilities in health research in East Africa: a mixed-method study protocol
Source: Wellcome Open Res. 2026 Feb 14;10:578. Originally published 2025 Oct 15. [Version 2] doi: 10.12688/wellcomeopenres.24916.2 (PMC12924008; doi:10.12688/wellcomeopenres.24916.2)
Supplement: Peer review history

# Reviewer report: Reviewer response for version 2

**DOI:** 10.21956/wellcomeopenres.28486.r147763

**Published:** 2026-02-25

**Authors:** Lionel Sakyi

**Affiliations:** 1 Centre for Migration Studies, University of Ghana, Accra, Ghana; 2 Global System for Mobile Communications, Accra, Ghana

All comments have been addressed. No further comment

Is the study design appropriate for the research question?

Yes

Is the rationale for, and objectives of, the study clearly described?

Yes

Are sufficient details of the methods provided to allow replication by others?

Partly

Are the datasets clearly presented in a useable and accessible format?

Yes

Reviewer Expertise:

Disability inclusion

I confirm that I have read this submission and believe that I have an appropriate level of expertise to confirm that it is of an acceptable scientific standard.

# Reviewer report: Reviewer response for version 2

**DOI:** 10.21956/wellcomeopenres.28486.r147765

**Published:** 2026-02-20

**Authors:** Janeth Aleman-Tovar

**Affiliations:** 1 California State University, Fresno, USA

Thank you for addressing my questions and concerns. I no longer have any reservations.

Is the study design appropriate for the research question?

Yes

Is the rationale for, and objectives of, the study clearly described?

Partly

Are sufficient details of the methods provided to allow replication by others?

Partly

Are the datasets clearly presented in a useable and accessible format?

Not applicable

Reviewer Expertise:

My research focuses on families of youth with disabilities and qualitative studies.

I confirm that I have read this submission and believe that I have an appropriate level of expertise to confirm that it is of an acceptable scientific standard.

# Reviewer report: Reviewer response for version 1

**DOI:** 10.21956/wellcomeopenres.27446.r141854

**Published:** 2026-01-07

**Authors:** Janeth Aleman-Tovar

**Affiliations:** 1 California State University, Fresno, USA

I had the pleasure of reading the manuscript entitled, “Disability Inclusive Youth (DIY) Research: An innovative and co-creative study to improve inclusion of children and youth with disabilities in health research in East Africa: A mixed method study protocol.” The study protocol describes the steps the research team plans to undertake to complete the longitudinal study. I have a few questions and comments listed below. I believe with appropriate changes, the manuscript is ready for indexing. 

**Study Approach**

 Component 1

 Participants: What are the inclusionary and exclusionary criteria for the 12 young people with disabilities? What will the practical research training consist of? What types of supports/accommodations will be provided to ensure the youth are successful? Provide some examples of reasonable (i.e., section study population…). Will there be specific criteria to determine if the participants are ready to participate (e.g., conduct the interview)? How was it determined that three months is sufficient time?

 How will the health researchers be selected (e.g., inclusionary and exclusionary criteria)?

 How did you determine 60 interviews were sufficient, and why were 6 (versus 10)  case studies selected? Also, how will the six case studies be selected?

 Component 2

 Is the fifth workshop and consultative meeting part of the four-day participatory workshop series? 

 How long are the workshops, and will they take place in-person or online?

 Component 3

 Who will conduct the interviews with the 12 youth health researchers with disabilities? 

 Some of my questions above have been answered in the following sections. You may want to add a note that refers the reader to the next sections regarding participant inclusionary criteria etc. 

**Study Design**

 The study consists of various steps; accordingly, I suggest that the authors create a flowchart or table outlining the procedures of the study to ease the reader's understanding. 

 Study Population and Participant Recruitment

 How will the research team determine that the selected youth participants are ready to conduct an interview? Will there be ways to measure their readiness?

**Data analysis**

 Ensure the section is all future tense (e.g., A thematic analysis approach was used...)

 Will the student researchers assist in data analysis? Who will analyze data?

**Limitations**

 I appreciate the authors acknowledging the limitations of the newly trained youth and believe that providing more information about their training is necessary. What is the timeframe for this study (e.g., when will each step be completed and how long will it take?)?

 Thank you for studying such an important topic. Good luck moving forward.

Is the study design appropriate for the research question?

Yes

Is the rationale for, and objectives of, the study clearly described?

Partly

Are sufficient details of the methods provided to allow replication by others?

Partly

Are the datasets clearly presented in a useable and accessible format?

Not applicable

Reviewer Expertise:

My research focuses on families of youth with disabilities and qualitative studies.

I confirm that I have read this submission and believe that I have an appropriate level of expertise to confirm that it is of an acceptable scientific standard, however I have significant reservations, as outlined above.

# Response

**Published:** 2026-01-15

**Authors:** Femke Bannink Mbazzi

**Affiliations:** Disability Research Group, MRC/UVRI and LSHTM Uganda Research Unit, Entebbe, Central Region, Uganda

We would like to thank the reviewer for the thoughtful review and comments.  In the study approach section under component 1 we have made reference to the various sections to ease reading. The in and exclusion criteria for the youth researchers are outlined in the study design section. We have described the content of the training under the study approach section under component one. We have added more detail on the type of accommodations provided under the ethical considerations section. The health researchers will be selected from academic and research institutions in Kenya, Rwanda and Uganda. A purposive sampling method will be used, enrolling 75 health researchers consecutively until the sample size is reached. The 15 health researchers with disabilities will be purposively selected through snowball sampling. Representation of gender and impairment will be ensured as much as possible. We have added detail in the study design section under the header study population and participant recruitment. We determined that a sample size of 60 youth with disabilities (approximately 20 per country) with representation of gender and impairment categories (physical, visual, hearing, cognition, communication and multiple) would be sufficient. The sample size is based on recommendations for qualitative studies and expected data saturation of groups of youth with different type of impairments with at least 4 in each impairment group. Purposive sampling will be used. From the in-depth interviews 6 youth will be purposefully selected. We chose 6 instead of case studies, to have 2 cases per country, a child and a youth. We will use photo voice and ask the children and youth to document their experiences in health research, for example clinic visits and intervention activities they participate in at home, at the health facility or in the community over a period of 3 months. To ensure rich data is collected, the youth researchers with guidance from the senior researchers will follow up closely with the participants during the data collection period. We plan to match 2 youth researchers with each case study participant and their family. We have clarified this under the study approach (component 1) and study design (study population and participant recruitment) sections. To develop guidelines and share best practices based on the study findings five participatory workshops will be held: 3 to design the guidelines in every country and create social media messages and story boards for the film and 2 multi-country meeting to finalize the guidelines and related messages. Each in country workshop will be a full day and take place in person. The intercountry workshops will have a hybrid format to allow key stakeholders who cannot be present in person to contribute to the design. We have clarified this further under the study approach section (component 2). The interviews with the 12 youth health researchers with disabilities will be conducted by a PhD student who will evaluate the youth researchers experiences. We have explained this in the study approach section (component 3). To clarify the study design, we have created a figure outlining the activities and timeframe of the study. After completing the research training and 3 months internships, the youth researchers will observe senior researchers conduct the first interviews with health researchers and key stakeholders. They will conduct mock interviews with researchers of the institutes they are affiliated to and will be evaluated for readiness by the senior research team member. Once ready, they will conduct their first interviews, whilst the senior researcher observes them and provides inputs where needed. After the youth researchers have completed at least two interviews satisfactory, they will start conducting interviews independently, still receiving feedback on the interview process from their supervisors through debriefing sessions. The initial training period is based on the DIY programme results from the pilot phase ( https://sjdr.se/articles/10.16993/sjdr.1307).  We have added this reference to the rationale section and added more explanation under the study approach section (component 1). We have ensured all text is written in the future tense. The youth researchers will assist in the data analysis. They will co-develop the codebook through an interactive workshop and will receive training in thematic analysis and the use of NVIVO for coding. All transcripts will be coded by the youth researchers with guidance from the research teams. A workshop to analyse the data together with be held after coding has been completed and youth researchers are expected to write draft analysis reports. They will also lead validation meetings with participants after the draft analysis is complete. This has been outlined in the study approach section (component 2).

 We have added information on the youth researchers training under the study approach section (component 2).

# Reviewer report: Reviewer response for version 1

**DOI:** 10.21956/wellcomeopenres.27446.r141856

**Published:** 2026-01-02

**Authors:** Lionel Sakyi

**Affiliations:** 1 Centre for Migration Studies, University of Ghana, Accra, Ghana; 2 Global System for Mobile Communications, Accra, Ghana

Summary of the protocol

 This protocol describes the Disability Inclusive Youth (DIY) Research, a mixed-method, longitudinal, co-creative participatory study in Uganda, Kenya, and Rwanda that aims to identify barriers and facilitators to including children and youth with disabilities in health research, co-design practical solutions and guidelines, and establish a regional disability knowledge and research centre. The study is organised into three components: participatory research led with youth co-researchers, guideline development through participatory workshops, and capacity building plus knowledge co-production/sharing. The rationale is grounded in African disability studies discourse, intersectionality, and Ubuntu framing, positioning peer support and youth leadership as central to shifting research culture and practice.

 Thank you for the opportunity to review this protocol paper. The protocol addresses an important gap: meaningful inclusion of children and youth with disabilities not only as participants but also as co-researchers and agenda-setters within African health research. That said, I have some few comments and suggestions that could strengthen the protocol below.

 I will not provide comments on the components, as I agree with all the review comments from the first reviewer.

 Study design

 The study is described as a longitudinal cohort study using a co-creative, participatory mixed-methods implementation research approach. The longitudinal element is clear for the 12 youth researchers, who are interviewed before, during, and after the programme. It is less clear whether the 60 youth participants and the 75 health researchers are interviewed over time or only once.

 The protocol states that the 60 youth participants will be recruited through academic and research institutions and other stakeholders, including organisations of persons with disabilities, using snowballing as needed. Because this approach may privilege youth already connected to institutions, the protocol should explain how the study will reach eligible participants who are less networked, especially rural youth, out-of-school youth, and those with communication or intellectual impairments.

 The protocol has internal inconsistencies in participant counts and wording that need to be resolved. For example, one section describes interviews with 75 health researchers plus 30 stakeholders, while another lists 60 interviews with health researchers plus 15 with disabilities (total 75). This may be correct, but the protocol needs one clear, consistent specification across all sections. Similar clarity is needed for the youth and caregiver interviews: the protocol refers to caregivers of children aged 0 to 8, children aged 8 to 17, and youth aged 18 to 30. Please state the expected number of interviews in each age group and clarify whether caregivers are interviewed separately, jointly, or within the same interview encounter.

 The youth co-researcher training and mentorship model is a key innovation, and the protocol outlines several elements (competitive selection, reasonable accommodations, a five-day training, monthly mentoring, annual intercountry meetings, and an initial three-month practical placement in ongoing projects). However, it should specify the training curriculum, including learning objectives, how competence will be assessed, safeguarding and emotional support arrangements, and the accommodations and resources guaranteed (for example assistive technology, transport, and interpreter time). This detail matters because cross-country data quality and consistency will depend heavily on how training and supervision are structured.

 For the photo and video voice case studies, the protocol describes participant training, three months of documentation, weekly follow-up, and narrative capture. It should also explain how privacy and third-party consent will be managed when filming or photographing in clinics and communities, and how the study will minimise inadvertent disclosure of disability status or research participation.

 The safeguarding plan includes staff training and referral pathways for disclosures of violence or acute health needs. Given the focus on youth and disability, add brief procedural detail on how referrals will operate in each country, how mandatory reporting duties will be managed, and how youth researchers will be supported when they encounter distressing narratives.

 The protocol permits caregiver involvement as proxy or assistant when impairment affects understanding or communication. This is pragmatic, but it raises ethical questions about voice, assent, and potential undue influence. A short section on how the study will prioritise youth assent and preference, manage private space for youth where feasible, and distinguish youth views from caregiver interpretations would strengthen the protocol. It is important to make their voice stand out.

 To support replicability, add more detail on the study tools and analysis where feasible. For example, summarise the main domains covered in the researcher survey and interview guides, and explain how quantitative and qualitative findings will be integrated to answer each research question. The qualitative approach is well specified; outlining the planned analysis for any survey or numeric data will complete the mixed-methods picture and make the integration strategy clear.

 Summarise the study design with a table or simple flow diagram. For example, include a table that lists each study component or research question alongside the data sources that address it (participant group and the planned number of interviews or activities). This would give readers a quick, coherent snapshot of the overall design without relying on dense narrative. A simple timeline figure showing key activities (training, data collection phases, workshops, and establishment of the knowledge centre) would also help.

 General comments:

- The protocol shifts between past and future tense, which makes it hard to tell what has already happened (for example, youth contributing to interview guide design and consenting). Perhaps the study could add a short “current status” paragraph and a simple timeline that states which activities are completed, ongoing, and planned (per country). This will also improve replicability and reduce ambiguity for readers.
- From a disability inclusion perspective, the protocol needs an explicit inclusion framework or set of auditable commitments. For example, it notes that youth contributed to interview guide design and data collection, but it does not specify which decisions youth researchers lead, which are shared, and which remain with senior academics (and why). Without this clarity, the project may appear inclusive while still reproducing hierarchies of expertise.
- Using the Washington Group Short Set for screening can support comparability, but the protocol should explain how it will address how screening will be interpreted alongside local understandings of disability. If applied too rigidly, a “moderate to severe” threshold could exclude people who self-identify as disabled or are disabled through social barriers but do not meet the cut-off, so this needs explicit attention.
- A practical way to strengthen disability inclusion is to make power and accountability commitments explicit in the design. For example, specify how youth researchers will be compensated and supported; what capacity building entails beyond a one-off training; how co-authorship will be handled; how disagreements between youth and senior researchers will be resolved; and what options youth have to decline activities without penalty.

Is the study design appropriate for the research question?

Yes

Is the rationale for, and objectives of, the study clearly described?

Yes

Are sufficient details of the methods provided to allow replication by others?

Partly

Are the datasets clearly presented in a useable and accessible format?

Yes

Reviewer Expertise:

Disability inclusion

I confirm that I have read this submission and believe that I have an appropriate level of expertise to confirm that it is of an acceptable scientific standard, however I have significant reservations, as outlined above.

# Response

**Published:** 2026-01-15

**Authors:** Femke Bannink Mbazzi

**Affiliations:** Disability Research Group, MRC/UVRI and LSHTM Uganda Research Unit, Entebbe, Central Region, Uganda

We would like to thank the reviewer for his positive and constructive review and helpful suggestions. In response to comments on the study design, we have made the following edits. The reviewer asked about the longitudinal aspect of the cohort study. The 12 youth researchers are interviewed before, during, and after the programme. The 60 youth participants and the 75 health researchers are interviewed only once. We have clarified this in the study design section. The 60 youth participants will be recruited through academic and research institutions and other stakeholders, including organisations of persons with disabilities, using snowballing as needed. The reviewer has raised an important concern about this approach privileging youth already connected to institutions. We hope to find eligible participants who are less networked, especially rural youth, out-of-school youth, and youth with communication or intellectual impairments through urban and rural community based organisations and community structures including local leaders and village and community health workers. We have added further details in the study participant section. We have reviewed the participant counts and ensured a consistent specification across all sections in the revised version: 75 health researchers of which 15 with a disability and 30 stakeholders. In addition, we have clarified that we aim to interview 60 children and youth disabilities, approximately 20 in each age group: 0-8 years, 8-17 years, 18 – 30 years in the study design section. We will only interview caregivers of children who are unable to express themselves in an interview.   We are grateful for the positive feedback on the innovative youth co-researcher training and mentorship model. We have further specified the training curriculum, including learning objectives, how competence is assessed, safeguarding and emotional support arrangements in the study approach section (component 1), and the accommodations and resources guaranteed (for example assistive technology, transport, and interpreter time) in the ethical considerations section.

 For the photo and video voice case studies, we have added how privacy and third-party consent will be managed when filming or photographing in clinics and communities, and how the study will minimise inadvertent disclosure of disability status or research participation, in addition to the description of participant training, three months of documentation, weekly follow-up, and narrative capture, in the study design section.

 The safeguarding plan includes staff training and referral pathways for disclosures of violence or acute health needs. We have added a brief procedural detail on how referrals will operate, how mandatory reporting duties will be managed, and how youth researchers will be supported when they encounter distressing narratives in the ethical considerations section in the ethical considerations section.

 The protocol permits caregiver involvement as proxy or assistant when impairment affects understanding or communication, as outlined by the reviewer. We agree it raises ethical questions about voice, assent, and potential undue influence. The study will prioritise youth assent and preference, manage private space for youth where feasible, and distinguish youth views from caregiver interpretations. One of the ways to mitigate parental involvement and ensuring voice is having youth with a disabilities conduct the interviews. It is often easier for them to ask to speak to the person privately and  which has been helpful in earlier studies conducted by study team members. We have added more detail on this in the study approach.

 To support replicability, the reviewer asked for more detail on the study tools and analysis. Whilst we were able to add some more details on the main domains covered in the researcher survey and interview guides, the tools are co-developed with the youth researchers and informed by each phase of the study. For example, after the initial interviews with health researchers and key stakeholders, the research team will develop the guides for the children and youth interviews and design the survey questions. Similarly the analysis approach on integrating quantitative and qualitative findings will be discussed with the youth researchers and defined during the research process. We have however added a few more, already known, details on the survey or numeric data in the study design section.

 In response to the reviewer’s request for a summary of the design with a simple flow diagram we have added a timeline figure showing key activities. In response to the reviewer’s general comments we have rewritten the paper in the future tense and added a timeline in the figure mentioned to indicate the different phases and current status. The reviewers suggests having an explicit inclusion framework or set of auditable commitments. We have tried specify this further in the revised protocol paper, specifically in the study approach and community engagement sections. The youth researchers and youth advisory groups contributed to interview guide design and are currently engaged in data collection. In the preparation phase they will participate in drafting the interview guide, selecting participants, contacting participants, setting up appointments, consenting participants and conducting interviews. With support from the study data manager they will participate in data storage, PTID labelling, transcribing data and conducted quality control on qualitative interview transcripts. They will also be trained in data analysis and actively participate in coding, thematic analysis, report writing and validation and dissemination of findings. We acknowledge the limitation of the Washington Group Short Set of defining a disability at moderate to severe threshold and plan to use this alongside participants own descriptions of their disability and allow for self-identification in addition. We have representatives of the National Councils for Persons with Disabilities on our advisory boards and consult them about in country disability definitions. We have clarified this further in the study design section. We made power and accountability commitments more explicit in the design. The youth researchers receive a monthly salary and reasonable accommodations. They have terms of reference and are part of the research team. The youth researchers have regular evaluation meetings with their supervisors and also have peer meetings to discuss challenges and find ways forward. Every quarter they participate in a cross-country exchange meeting with the study team and in country they participate in weekly study team meetings. They also take part in other meetings of the organisations they are employed by if relevant to their development and work. Examples of provided reasonable accommodations include a sign language interpreter for youth researchers with a hearing impairment, captioning of all meetings, breaks and time of work for youth with psychosocial disabilities, a personal aid for a youth researcher with multiple impairments, transport arrangements and screen read programmes for youth researchers with visual impairments. Disagreements will be resolved following institutional human resource a conflict resolution procedures. Similarly, access to external counselling support is available for all staff. We have added some more information on this in the ethical considerations section. The youth researchers receive trainings on a quarterly basis, focused on topics relevant to the stage of the study, e.g. on tools design and transcriptions were conducted in the first year of the study, in the second year training on NVIVO coding and data analysis will be provided, whilst further training on writing publications and presentation skills will be offered in the third year. In addition, youth researchers have access to staff training opportunities within their respective institute. Further details have been provided in study approach section.

# Reviewer report: Reviewer response for version 1

**DOI:** 10.21956/wellcomeopenres.27446.r137128

**Published:** 2025-11-13

**Authors:** Tess Bright

**Affiliations:** 1 The University of Melbourne, Melbourne, Australia

- Thank you for the opportunity to review this important paper. How to include young people with disabilities as co-researchers in research that is about them is an valuable contribution to the literature, particularly in the context of limited research on this topic in the African setting. I have some suggestions and clarifying questions for authors below.
- Component 1 is a bit sparse on detail
  - What the training entailed and who conducted the training?
  - Who are the stakeholders? What type of health researchers?
  - How many of each age group of people with disabilities will be interviewed? Any consideration of intersections with other elements of identity? Such as gender and location (rural etc)?
  - What will the case study development entail ?
- Component 2
  - Not clear who attended workshops in each country
  - Which guidelines were reviewed in the workshops? How were these identified?
  - Children and young people are quite different groups with different needs and different ethical considerations – how are you going to capture this in the guidelines? More consideration is needed on some existing approaches to including children and young people in research (with or without disabilities)
  - Not clear exactly what the purpose of the guidelines are – are they for health researchers looking to do inclusive research with young people with disabilities? Or are they for young people with disability who want to do health research?
- Component 3
  - How are the Youth Advisory Groups formed
  - Will you be doing more than ‘consult’ people with disabilities –
    - “This may include the establishment of a consultative group or a pool of youth with disabilities from which researchers can select to provide inputs for grant applications and implementation of health research programs in the future.” – instead of ‘select’ here, suggest you could say ‘work with’ or something similar that suggests youth with disabilities have a say in what they take part in
    - Suggest you could mention co-designing grant applications that address concerns of young people with disabilities and leadership of people with disability in research
- Study design
  - 75 health researchers, 15 with disabilities, will answer a survey questionnaire and will be interviewed. – justify why 15 with disabilities
  - A lot of different data is being collected and sometimes it is hard to follow, could this be better captured in a table or a figure to show the different participants and purposes of each component?
- General comments
  - Authors should choose one tense throughout. I understand some components have been done, and some are planned, but changing between past and future tense impacts the flow
  - Some typos throughout which should be carefully checked
  - A lot of detail is repeated throughout and could be consolidated or put in to tables to improve flow and understandability
  - How will power imbalances be addressed between ‘established researchers’ and co-researchers with disability?

Is the study design appropriate for the research question?

Yes

Is the rationale for, and objectives of, the study clearly described?

Yes

Are sufficient details of the methods provided to allow replication by others?

Partly

Are the datasets clearly presented in a useable and accessible format?

Yes

Reviewer Expertise:

Disability

I confirm that I have read this submission and believe that I have an appropriate level of expertise to confirm that it is of an acceptable scientific standard, however I have significant reservations, as outlined above.

# Response

**Published:** 2026-01-15

**Authors:** Femke Bannink Mbazzi

**Affiliations:** Disability Research Group, MRC/UVRI and LSHTM Uganda Research Unit, Entebbe, Central Region, Uganda

Thank you for reviewing our paper and the helpful suggestions. Under the study approach section (component 1) we have elaborated on the content and facilitation of the training. We have also clarified (under study design, study population and participant recruitment) that stakeholders include regulatory bodies, national research councils, national councils for persons with disabilities, organizations of persons with disabilities and government officials. Health researchers are researchers who are actively involved in conducting health research in academic or other institutions in East Africa, we aim to interview research investigators as well as implementers. We have clarified that we aim to interview 60 children and youth disabilities, approximately 20 in each age group: 0-8 years, 8-17 years, 18 – 30 years. We will consider the intersections with gender, location and poverty by recruiting a similar number of female and male participants, rural and (semi) urban areas, from different economic backgrounds. Participants for the case studies will be selected from the 60 children and youth interviewed, we will ensure representation of gender and disability and participation in different types of health research. In the case studies, children and youth with disabilities will narrate their experiences about participating in a research study. They will use photo voice, or audio voice if visually impaired, to document their experiences. Guiding questions and training on the use of photo or audio voice will be developed with the youth researchers and research team members after the qualitative interview data has been collected. We have added further details in the study design section. Under the study approach section (component 2) we have clarified that we will have participatory workshops in each country to develop the guidelines for inclusive practices in health research in East Africa. These workshops will be attended by the youth researchers and research teams, youth advisory group members, and selected key stakeholders, including regulatory bodies and representatives from the Ministry of Health. In preparation of the workshops, the youth researchers will review existing approaches to include children and young people in research, together with the study teams. These approaches will be summarized, together with key findings from the data collection from each country to inform the discussions and development of guidelines in each country. The guidelines are for health researchers to make their research more inclusive of young people with disabilities.    Youth with disabilities from Rwanda, Kenya, and Uganda are invited to participate in the DIY Youth Advisory Groups (YAG) for this study. In Uganda, the existing Youth Advisory Group at the MRC/UVRI & LSHTM Uganda Research Unit will be asked to volunteer participants for the advisory meetings specific to the DIY study. In addition, youth representatives who participated in the completed Disabled Youth Investigates study, which informed the design of the DIY study, will be invited. In Kenya and Rwanda YAG members will recruited in consultation with the National Council for Persons with Disabilities and organizations of persons with disabilities. The organizations will be asked to nominate members who represent a diverse range of impairments, age groups, and genders. We have further clarified this under the community engagement section. We have rephrased the text on the consultative group in the study approach section (component 3). Youth with disabilities will co-design and offer guidance and advice for health researchers on grant applications and implementation of health research programs in the future. In total 75 health researchers will be interviewed, 25 in each country. We expect to learn more from all health researchers about their experiences and challenges with including children and young persons with disabilities in health research. In addition, we hope to interview 5 health researchers with a disability in each country, to share their experiences as disabled researchers and learn from approaches they have found successful. We believe having 15 interviews focused on this will be sufficient to gain insights, whilst also acknowledging that there are very few health researchers with a disability in East Africa currently. We have added a figure to show the different participants and data collection methods. We have changed the tense to future tense and included a timeline in the figure to indicate which activities will be conducted at certain timepoints. Power imbalances between established researchers and young co-researchers with disability will be addressed through participatory and co-production approaches that embed co-researchers in all stages of the study, including co-designing research questions, data collection tools, and contributing to data collection and analysis. Shared decision-making will be operationalised through youth led meeting facilitation, mentoring, ongoing reflexive practices, including youth researcher reflexive journals, regular feedback sessions and evaluations with established researchers. A PhD student will specifically study the experiences of youth researchers; power dynamics will be one of the areas of focus. We have clarified this in the study approach section of the paper
